# Macroalgae Bioplastics: A Sustainable Shift to Mitigate the Ecological Impact of Petroleum-Based Plastics

**DOI:** 10.3390/polym16091246

**Published:** 2024-04-29

**Authors:** Nehal E. Elkaliny, Nurah M. Alzamel, Shaaban H. Moussa, Nour I. Elodamy, Engy A. Madkor, Esraa M. Ibrahim, Mostafa E. Elshobary, Gehan A. Ismail

**Affiliations:** 1 Botany and Microbiology Department, Faculty of Science, Tanta University, Tanta 31527, Egypt; 2 Department of Biology, College of Science and Humanities, Shaqra University, Shaqra 11961, Saudi Arabia

**Keywords:** macroalgae, bioplastic, biodegradability, petroleum-based plastics, ecological impact, applications

## Abstract

The surge in global utilization of petroleum-based plastics, which notably heightened during the COVID-19 pandemic, has substantially increased its harm to ecosystems. Considering the escalating environmental impact, a pivotal shift towards bioplastics usage is imperative. Exploring and implementing bioplastics as a viable alternative could mitigate the ecological burden posed by traditional plastics. Macroalgae is a potential feedstock for the production of bioplastics due to its abundance, fast growth, and high cellulose and sugar content. Researchers have recently explored various methods for extracting and converting macroalgae into bioplastic. Some of the key challenges in the production of macroalgae bioplastics are the high costs of large-scale production and the need to optimize the extraction and conversion processes to obtain high-quality bioplastics. However, the potential benefits of using macroalgae for bioplastic production include reducing plastic waste and greenhouse gas emissions, using healthier materials in various life practices, and developing a promising area for future research and development. Also, bioplastic provides job opportunities in free enterprise and contributes to various applications such as packaging, medical devices, electronics, textiles, and cosmetics. The presented review aims to discuss the problem of petroleum-based plastic, bioplastic extraction from macroalgae, bioplastic properties, biodegradability, its various applications, and its production challenges.

## 1. Introduction

The increased use of plastic materials in the modern world, especially during the COVID-19 pandemic, has resulted in the accumulation of huge amounts of plastic waste in various ecosystems, leading to negative impacts on the environment and human health [1,2]. Petroleum-based plastics, which constitute the majority of plastics used today, are non-biodegradable. This leads to the persistence of plastic waste in the environment for hundreds of years [3]. Consequently, there has been a growing interest in developing sustainable and biodegradable alternatives to petroleum-based plastics. Bioplastics, as biodegradable and renewable alternatives to conventional petroleum-based plastics, have gained significant attention in recent years due to their environmental benefits [4]. The realm of biobased plastics is a diverse landscape encompassing many methods and materials, each offering unique attributes for creating sustainable alternatives. Primarily, bioplastics find their foundation in polysaccharides, proteins, and lipids, each contributing distinct features and characteristics that render them suitable for various sectors. The crux of bioplastics lies in their origin—derived from renewable materials and ushering in an eco-friendlier approach to plastic production. A plethora of biomaterials is already harnessed to manufacture bioplastics, including but not limited to, corn, potatoes, potato peels, sugar cane, banana peels, agricultural waste, algae, vegetable oils, wood, food waste, and various cereal crops [5]. This extensive range of sources not only underscores the versatility of bioplastics but also aligns with the overarching goal of sustainability in material sourcing.

Currently, the landscape of bioplastics is dominated by starch-based variants, followed closely by polylactic acid (PLA), poly-3-hydroxybutyrate (PHB), polyamide 11 (PA 11), and organic polyethylene (PE) [6]. Each type exhibits its own set of characteristics, making them suitable for diverse applications across industries. However, a notable and promising innovation on the horizon is the development of bioplastics crafted from seaweeds (macroalgae). This latest advancement demonstrates the ongoing evolution in sustainable materials and holds the potential to contribute significantly to the environmental footprint of the plastic industry. As research and innovation continue to advance, biobased plastics continue to grow, offering a more sustainable future for the materials that play a pivotal role in our daily lives.

Seaweeds are photosynthetic organisms thriving in marine and freshwater environments. They are classified based on their color, namely green (Chlorophyta), red (Rhodophyta), and brown (Phaeophyta). Seaweeds have diverse applications, including medicine, cosmetics, fertilizers, biofuel, wastewater treatment, paper production, aquaculture, plastic manufacturing, and more [7,8,9,10,11,12,13]. Macroalgae boast unique advantages for bioplastic production, setting them apart from other feedstocks [14]. Their appeal as a renewable and sustainable bioplastic source is evident in various aspects. Macroalgae exhibit a remarkable growth rate, generating a substantial biomass per unit area compared to terrestrial plants, which translates into cost-effective bioplastic production [15]. This high productivity makes macroalgae a cost-effective feedstock for bioplastic production. Algae typically contain less than 5% lignin, which simplifies the breakdown process during bioplastic production. Unlike the high lignin content (up to 35%) of plants’ cell walls [16], the reduced lignin content in macroalgae minimizes the energy and cost required for extracting the polysaccharides essential for bioplastic production [17]. Moreover, macroalgae act as potent carbon sinks by absorbing carbon dioxide at a rate up to 20 times higher per unit area than terrestrial plants. This capacity not only contributes to climate change mitigation but also contributes to reducing greenhouse gases. This capacity also aligns with circular economy principles by offering a means to reduce the carbon footprint of the plastic industry [18,19]. Algae-derived bioplastics have plenty of advantages including, renewability, biocompatibility, and biodegradability, making them sustainable alternatives to traditional plastics [20]. They have high mechanical strength, which facilitates their processing into a variety of shapes, sizes, and textures, making them suitable for use in a wide range of products [1,21]. Many studies have recommended macroalgae-derived bioplastics for plenty of applications, including packaging, medical devices, textiles, cosmetics, and drug coatings [22]. Macroalgae bioplastics are water and UV-light resistant, making them suitable for outdoor applications [23]. The enclosure of seaweed polysaccharides into biofilm for active packaging formulations can provide protection for the product against bacteria, oxidation, and UV rays, which improve the safety and shelf-life properties of the product [24]. Thus, macroalgal bioplastics are promising for further research in the field to develop a sustainable and eco-friendly alternative to fossil plastics with several desirable properties [24].

In light of the previous feedback, this review article seeks to furnish an extensive overview of macroalgae-based bioplastic production. It will delve into the extraction methods pivotal in converting macroalgae biomass into bioplastics, exploring potential applications and thoroughly examining the environmental and economic implications of macroalgae-based bioplastics.

## 2. Plastic Pollution and Its Significant Detrimental Impacts

Plastic pollution is the accumulation of plastic matter and particles in the Earth’s environment that harmfully affects humans, biota, and their habitat. Currently, the annual output of plastic stands at approximately 450 million tons, and it is expected to undergo a twofold increase by 2045. Inefficient collection and processing of plastic waste contribute to the pervasive presence of plastic fragments in the environment. Regrettably, a significant majority—three-quarters—of all plastic produced ultimately becomes waste. A mere 9% undergoes recycling, 12% is incinerated, and a staggering 79% is either relegated to landfills or released into the ecosystem [25]. Once plastics are discarded into the environment, abiotic stresses fragment them into smaller pieces, accumulating them for extended periods of hundreds or thousands of years [2]. Plastic fragments can be categorized into three main types: macro, micro, or nano debris. Macroplastics are relatively large plastic debris that can be visually identified without magnification. These include plastic bottles, bags, packaging materials, fishing nets, and other larger plastic objects. The diameter or size of macroplastics can range from a few centimeters to several meters, depending on the specific objects [26].

Microplastics are small plastic particles or fragments <5 mm (0.2 inches) in size. Microplastics can be further classified into two categories: primary microplastics and secondary microplastics, in addition to nanoplastics [27,28]. Primary microplastics are purposefully manufactured small plastic particles, often used in facial scrubs, toothpaste, and cleaning agents. Secondary microplastics are formed by breaking more oversized plastic items through UV, temperature, and wave action processes. These include particles from plastic bags, bottles, and other plastic debris that gradually degrade into smaller pieces [27,28].

**Figure 1:**
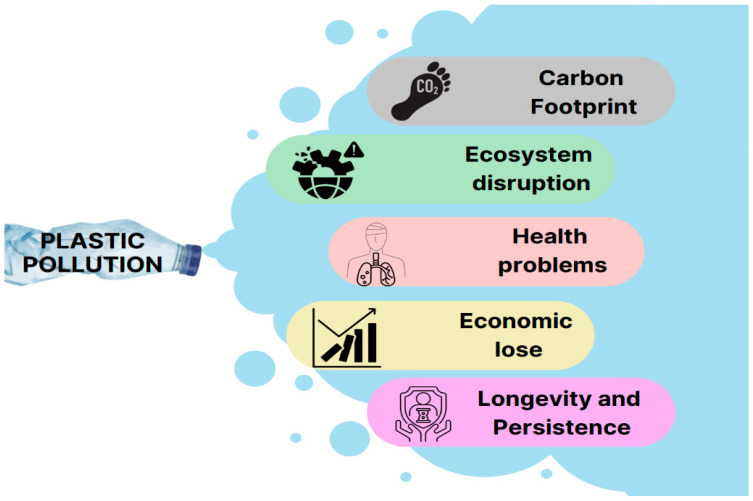
Plastic pollution and its significant detrimental impacts.

Nanoplastics are the most miniature form of plastic debris at <1 micrometer (0.001 mm) in size. They are the result of the further degradation and breakdown of microplastics. Nanoplastics are hard to detect and describe due to their extremely tiny size [29]. All three types of plastic debris pose environmental concerns, as they can persist in ecosystems, accumulate in wildlife, and potentially enter the food chain. Their widespread presence has raised concerns about the long-term effects on ecosystems and human health [30]. Therefore, nanoplastics’ toxic effect increases yearly and covers vast environmental fields [31]. This multifaceted impact can be distilled into five major categories, as shown in Figure 1.

### 2.1. Carbon Footprint

Plastics, primarily sourced from fossil fuels, exhibit a significant environmental impact by emitting greenhouse gases (GHGs) at various stages of their life cycle. This environmental footprint extends from the initial extraction of raw materials to the ultimate disposal at their end-of-life (EOL). The production, utilization, and disposal of plastics contribute to the release of GHGs, playing a role in the broader discourse surrounding climate change. Plastic production has a substantial carbon footprint that exacerbates climate change. The raw materials for most plastics are derived from fossil fuels like oil, gas, and coal. Extracting these hydrocarbons via activities like fracking and drilling generates emissions of carbon dioxide and methane [32]. The polymerization process to turn raw fossil resources into plastic resin also consumes large amounts of energy, often sourced from fossil fuels. The heat, pressure, catalysts, and transportation involved in plastic manufacturing further increase GHGoutputs [33].

Overall, the carbon footprint of plastics begins with the extraction of non-renewable fossil inputs and continues through energy-intensive production processes. This print persists long after end use as waste plastic releases emissions during decomposition and incineration. It is estimated that global plastic production and after-use accounts for 3.8% of annual fossil fuel usage and contributes over 850 million metric tons of GHGs annually [32]. The proliferation of plastic represents a significant contributor to anthropogenic climate change. Tackling plastic pollution by reducing unnecessary production and replacing fossil feedstocks with renewables can mitigate environmental damage. However, steep reductions will be required to curb plastics’ carbon footprint and align with climate change mitigation targets.

### 2.2. Ecosystem Disruption

Plastic pollution severely impacts wildlife, particularly marine animals, and birds. They can mistake plastic debris for food or become entangled in plastic items like fishing nets, causing injuries or death. Moreover, larger animals may ingest smaller organisms that have consumed microplastics, leading to bioaccumulation and potential harm to their health [34]. Plastic waste entering water bodies can significantly disrupt natural processes and the ecological balance of aquatic environments. Once plastics accumulate in lakes, rivers, and oceans, they can interfere with water circulation patterns that transport nutrients and oxygen to organisms [35]. Floating plastic debris forms dense surface layers that block sunlight penetration into deeper waters, reducing photosynthesis by aquatic plants [36]. Sinking plastics also smother benthic sediments, habitats, and nesting sites [37]. These physical impacts cascade through ecosystems. Alterations to circulation, light, and habitats affect the growth and survival of aquatic plants like seagrasses and algae that form the base of food chains [38]. Declines in these primary producers influence populations of zooplankton, fish, and other consumers dependent on them for sustenance and shelter [39]. In addition, plastics can leach chemical additives that trigger endocrine, immune, and neurotoxic effects in marine life [36]. The accumulation of plastic waste has been linked to decreases in species diversity and richness as sensitive organisms perish due to chemical and physical disturbances [40]. Invasive species more tolerant of pollution may proliferate, further changing community composition [37]. Removing keystone species that play central ecological roles can also destabilize ecosystems [41]. By degrading habitats, altering food webs, and enabling invasives, plastic pollution significantly threatens the biodiversity, resilience, and balance of aquatic systems [42,43,44].

### 2.3. Health Problems

Plastic can indirectly affect human health through various pathways. Plastic debris can release harmful chemicals into the environment, causing food and water contamination. When plastic particles or microplastics are ingested by marine life, they can enter the human food chain through seafood consumption [45]. Microplastics have been found in water sources, bottled water, and even in the breath air [45,46]. When plastic waste is burned, it releases toxic gases and particulate matter into the air, contributing to air pollution and respiratory problems [47]. Also, certain plastics contain toxic chemical additives like bisphenol A and phthalates. These chemicals can leach into food, beverages, and personal care products, potentially disrupting hormonal balance and leading to adverse health effects [48,49].

According to a recent study, a new technique has found microplastic particles in human organs, including the brain. The researchers added particles to 47 lung, liver, spleen, and kidney tissue samples obtained from a tissue bank established to study neurodegenerative diseases [50]. Their results showed that the microplastics could be detected in every sample. The impact of microplastics on human health is not yet known. Still, it is concerning that these non-biodegradable materials that are present everywhere may enter and accumulate in human tissues. The discovery of microplastics in human organs is a relatively new field of research, and scientists are still trying to understand their potential health risks. However, microplastics have polluted the entire planet, from Arctic snow and Alpine soils to the deepest oceans. People are also known to consume them via food and water and to breathe them in [27,49].

### 2.4. Economic Lose

Plastic pollution has detrimental economic effects that manifest at both local and global scales. Governments, environmental organizations, and communities dedicate substantial financial resources and time toward cleaning up plastic waste from beaches, waterways, and other public spaces. These efforts require mobilizing staff, equipment, transport, and waste disposal infrastructure, amounting to high costs [51]. For example, the state of California spends over $500 million annually removing trash, much of it plastics, from coastal areas alone [52]. As mentioned above, plastic pollution can affect tourism and recreational activities by damaging coastal and marine environments. It can also impact fisheries and aquaculture industries, leading to economic losses for communities dependent on these sectors [53]. One study found marine litter reduced revenues of coastal municipalities in the Mediterranean by 5% through lowered tourism demand [54]. Plastic pollution further threatens fisheries and aquaculture, which are critical to many regional economies. Floating plastics can snag fishing gear and aquatic farms, while microplastics ingested by fish affect their commercial value [53]. The Asia-Pacific Economic Cooperation estimates plastic pollution costs $10.8 billion in losses annually for its member states in the fishing and aquaculture sectors [55]. These direct cleanup expenses and economic impacts on tourism, recreation, and marine industries have detrimental spillover effects on small businesses and community livelihoods. Tackling plastic pollution is crucial not just for the environment but also for socio-economic resilience worldwide.

### 2.5. Longevity and Persistence

One of the key challenges of plastic is its longevity in the environment. Plastics can take hundreds of years to decompose naturally, leading to a persistent and long-lasting pollution problem. This means that the plastic waste generated today will continue to impact ecosystems and human health for generations to come [56]. Addressing the effects of plastic pollution requires a multifaceted approach, including reducing plastic production and consumption, improving waste management practices, and increasing recycling rates [57]. Additionally, efforts to promote biodegradable and compostable plastics, along with alternative materials, are underway to reduce plastic waste and its environmental impact [58]. Among the recent solutions to overcome the hazards of using conventional plastics is developing more natural and economical types of bio-based plastics. The pervasive and detrimental impacts of plastic pollution, as outlined in this section, underscore the urgent need for sustainable alternatives to traditional plastics. Fortunately, recent advancements in the field of bioplastics offer a promising solution to mitigate the environmental challenges posed by plastic waste. In particular, the use of macroalgae as a raw material for bioplastic production presents a viable and environmentally friendly approach.

## 3. Environmental and Economic Benefits of Macroalgae Bioplastics

Macroalgae bioplastics hold immense promise in delivering a dual impact on the environment and economy. Firstly, using macroalgae as a raw material proves to be a cost-effective alternative compared to other bioplastic sources, mitigating the rising expenses associated with bioplastic production [59]. This economic advantage promotes affordability and contributes to overall cost reductions in the bioplastics industry. Secondly, the production of macroalgae bioplastics creates a ripple effect in job creation. Beyond the bioplastics sector, this extends to the agriculture and aquaculture industries, fostering employment opportunities and supporting a robust economy [60]. Lastly, the growing market demand for sustainable and eco-friendly products positions macroalgae bioplastics is a key player in meeting consumer preferences. As environmental consciousness continues to rise, the demand for eco-friendly alternatives is expected to surge, creating a conducive market for macroalgae bioplastics [6]. This confluence of cost-effectiveness, job creation, and market alignment underscores the potential of macroalgae bioplastics as a sustainable and economical solution for environmentally friendly materials. Moreover, the sustainability of the formed bioplastics is a basic attention for researchers. The term life cycle assessment (LCA) means the study of the advantages and disadvantages of bioplastics under usage as an alternative to regular plastics [4]. LCA is a standard methodology with ISO 14040 [61] and 14044 [62] that can examine the socio-economic and environmental effects of goods [4]. Social LCA includes the manufacture, distribution, use, and removal of specific raw materials or products that can cause negative effects from a social standpoint. The term life cycle costing (LCC) denotes the entire costs during the life cycle of a product. The (EOL) recycling of several bio-based polymers was also discussed [63,64]. These terms indicated that bioplastic should be efficiently sustainable and applicable for mechanical, chemical, or biological recycling. The gap in the present research comparing fossil-based polymers and bio-based polymers is due to the lack of LCA comparable studies data and the absence of uncertain analysis. Most studies ignored the hazards of using additives during bioplastic formulation and their potential leakage into the environment or emergence of biogenic CO_2_ in the air and its impact on the growing crops.

### Bioplastic Industry: Future Prospective for Economic Growth and Job Creation

The bioplastic industry is a fast-growing sector that has significant potential for global economic development and job formation. Government regulations are aimed at reducing plastic waste and advancements in bioplastic technology [65]. Traditional plastic packaging is a significant contributor to plastic waste and pollution. Bioplastics offer an alternative that is more environmentally friendly and can help reduce the amount of plastic waste that ends up in landfills and oceans. As consumers become more aware of the environmental impact of plastic packaging, they are demanding more sustainable alternatives, creating a significant opportunity for bioplastics manufacturers [66,67]. In addition to packaging, bioplastics are also used in a variety of other life fields, including textiles, electronics, automotive parts, and medical equipment [68,69,70]. This diversification of uses is another factor driving growth in the bioplastic industry. As bioplastics become more widely used in different sectors, their demand is likely to continue to increase [71].

Another factor is government regulations aimed at reducing plastic waste. Many countries and regions around the world have implemented or are considering bans or restrictions on single-use plastics, such as bags, straws, and cutlery [72,73]. These regulations create a significant opportunity for bioplastics manufacturers. They offer an alternative that can help businesses comply with the regulations while reducing their environmental impact [74,75]. Advancements in bioplastic technology are also motivators for this industry. As research and development efforts continue, new and innovative bioplastic materials with improved properties and performance are being developed and can be used in a wider range of applications [58,76]. Furthermore, the development of the bioplastic industry has significant potential for new job opportunities. As the industry grows, there will be a need for skilled workers in areas such as research and development, manufacturing, and marketing [77]. By the way the industry continues to evolve, there will be a need for researchers and scientists to explore new avenues of innovation, and business persons to bring new products to market. These workers will need to have a deep understanding of materials science and expertise in areas such as biotechnology, chemistry, and engineering. Manufacturers will need to ramp up production to meet the increased request. This will require skilled workers in areas such as machine operation, quality control, and logistics [58].

## 4. History of Bioplastic

The history of bioplastics dates back several decades and has seen significant developments and advancements over time. The concept of using renewable resources to produce plastics can be traced back to the early 20th century. In the 1920s, researchers began experimenting with materials such as cellulose and starch to develop bio-based plastics [78]. The first commercial bioplastics emerged in the 1970s. PLA, derived from corn starch, was a notable example and was introduced as a biodegradable and compostable alternative to traditional plastics [79]. Throughout the 1980s and 1990s, there were significant advancements in biopolymer technology. Researchers explored various renewable resources and developed processes to convert them into bioplastics. New biopolymers, such as polyhydroxyalkanoates (PHAs), were also introduced [80]. By the early 2000s, bioplastics started to gain attention as more sustainable alternatives to fossil plastics. They found applications in various industries, including packaging, automotive, agriculture, and consumer goods. Companies began producing bioplastic products on a larger scale [81].

In recent years, there has been a growing interest and innovation in bioplastics. Researchers and companies are exploring new materials and technologies to improve bioplastics’ performance, biodegradability, and environmental impact. Bioplastics derived from algae, bacteria, and waste materials are being developed [81]. The future of bioplastics looks promising, with ongoing research and development focused on improving their properties, reducing costs, and expanding their applications. The goal is to create more sustainable and environmentally friendly alternatives to conventional plastics [80].

## 5. Classification of Bioplastics

Bioplastics can be classified based on their origin, composition, and biodegradability. Bio-based or renewable resource-based bioplastics are derived from renewable resources such as plants, crops, or algal biomasses. They are made from bio-based polymers from corn, sugarcane, potatoes, or cellulose from wood pulp [5,82]. Another category is the biodegradable or compostable bioplastics. These polymers are designed to break down into simpler components under natural processes or specific conditions, such as exposure to heat, moisture, or microorganisms. Compostable bioplastics are a specific type of biodegradable plastic that breaks into organic matter in a composting environment, leaving no harmful residues [82,83]. It is important to know that not all bioplastics are biodegradable, and not all fossil plastics are non-biodegradable. The terms “bioplastic” and “biodegradable” are not synonymous. Understanding the specific characteristics and EOL options for each type of bioplastic is crucial for sustainable plastic usage and waste management [84].

## 6. Macroalgae as Sources of Bioplastic Compounds

Macroalgae, also known as seaweeds, encompassing Rhodophyta, Phaeophyta, and Chlorophyta, exhibit diverse forms and sizes in response to varying seawater depths. Their classification based on pigmentation, notably red, brown, and green algae, provides a comprehensive framework for understanding their rich biodiversity [6]. From filamentous and leafy structures to calcareous forms, the morphology of macroalgae is a testament to their adaptability. These versatile organisms inhabit a spectrum of marine environments, ranging from intertidal zones to profound depths. The intricacies of their shapes and sizes are influenced by factors such as temperature, light, salinity, pollution, nutrients, and water currents [81]. The physiological and ecological adaptations of macroalgae to these environmental variables and grazing pressures contribute to their distinct distribution within habitats. They can vastly grow in a variety of marine environments and harvested sustainably. Additionally, they do not require any arable land or freshwater to grow, making them a sustainable alternative to land-based crops. Macroalgae can be harnessed from two primary origins: natural marine ecosystems and controlled aquaculture systems [18]. Macroalgae are a promising less exploited raw material, which has a rich content (25–60%, *w*/*w*) of carbohydrates, polysaccharides or hydrocolloids. Compared to lignocellulosic biomass, hydrolysis of macroalgae occurs under milder conditions due to their very low lignin content, resulting in hydrolysates that are pentose-poor [6]. However, the polysaccharides in each seaweed group are different in chemical properties, contents, and functions in relation to the seaweed origin, species, cultivation conditions, and extraction process. Understanding seaweed habitat preferences and adaptability can pave the way for sustainable exploitation and utilization of these bioresources in various applications, including as a potential source of bioplastics [6]. Some common seaweed genera have been used efficiently to make bioplastic films such as *Ulva*, *Codium*, and *Enteromorpha* for green seaweeds, *Kappaphycus*, *Eucheuma*, and *Gracilaria* for red seaweeds, and *Ascophyllum*, *Laminaria*, and *Lessonia* for brown seaweeds [23,24].

### 6.1. Main Compounds of Bioplastics Produced from Brown Macroalgae

Brown macroalgae, or Phaeophyta, thrive in shallow coastal waters globally, distinguished by their brownish hue attributed to fucoxanthin pigments [85]. Abundant in polysaccharides, they serve as a valuable resource for bioplastic production [86]. Phaeophyta species synthesize and accumulate many secondary metabolites that can be used as promising sources of bioplastic production as will be discussed in the following.

#### 6.1.1. Alginate-Based Bioplastics

**Figure 2:**
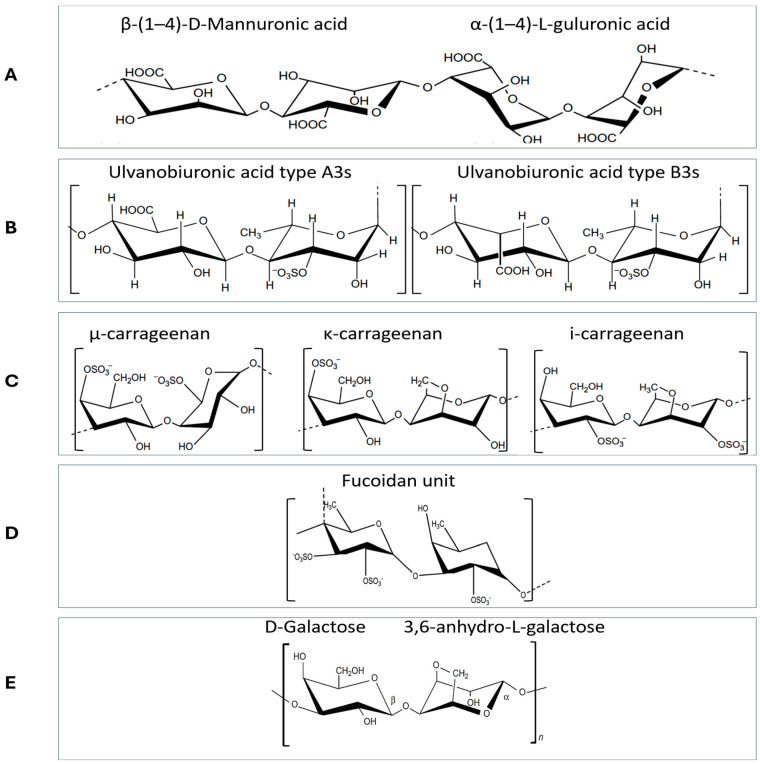
Chemical structures of different polysaccharides extracted from macroalgae: (**A**) Alginate, (**B**) Ulvan, (**C**) Carrageenan, (**D**) Fucoidan, and (**E**) Agarose [97].

Alginate is a natural polysaccharide extracted from brown seaweeds as a derivative of alginic acid and its salts. Alginate has been widely used to develop biodegradable plastic films and gels. The polymer consists of two blocks, β-(1 → 4)-D-mannuronic and α-L-guluronic acid, which make up alginates in a variety of different M/G ratios (Figure 2) [87]. A high content of guluronic acid caused robust and more elastic gelling properties. Alginates are highly hydrophilic, so it is crucial to mix them with other elements for more water resistance when in contact. The presence of this polysaccharide as an insoluble calcium or magnesium salt in the cell matrix gives the tissue its flexibility and sturdiness [88]. Alginate-based bioplastics are biodegradable, nontoxic, and have good mechanical properties as thickening and stabilizing agents. Therefore, they are used in food packaging, edible films, agriculture, and medicine [89].

#### 6.1.2. Fucoidan-Based Bioplastic

Fucoidan, a polysaccharide found in brown algae, contains different percentages of l-fucose and sulfate ester groups based on the species (Figure 2) [90]. It can be used as a feedstock for the production of alginate, a biopolymer that has applications in the food and pharmaceutical industries [91].

#### 6.1.3. Laminarin-Based Bioplastics

Laminarin is a low molecular weight β-glucan storage polysaccharide present in the cell wall of brown algae. Laminarin-based bioplastics are biodegradable and nontoxic. Laminarin can be hydrolyzed into glucose monomers, which can be used as a feedstock for producing PLA, a biodegradable polymer [92]. The production of PLA from laminarin involves two main steps: hydrolysis and fermentation. Yeast enzymatic hydrolysis breaks down the laminarin into glucose monomers, while bacterial fermentation converts the glucose monomers into lactic acid, which is then polymerized to form PLA [93]. They can be used in packaging, agriculture, cancer therapies, drug/gene delivery, tissue engineering, antioxidants, and anti-inflammatory functions [94].

### 6.2. Main Compounds of Bioplastic Produced from Red Algae

Red algae, or Rhodophyta, are diverse eukaryotic organisms that inhabit freshwater lakes and marine environments, showcasing a distinctive red color from phycoerythrin, carotenoids, and chlorophyll a pigment [95]. Their starch and galactan sulfate polymer content positions red algae as a viable feedstock for bioplastic production [96]. The structure of different seaweed polysaccharides is illustrated in Figure 2.

#### 6.2.1. Carrageenan-Based Bioplastic

Red seaweed contains different kinds of sulfated polysaccharides, such as carrageenan, agar, and agarose [98,99,100,101,102]. Carrageenan is a linear sulfated polysaccharide present in the cell wall and intercellular matrix of various species of red seaweeds. Several isomers of carrageenan are known as κ-, λ-, and ι-carrageenan (κ-kappa, λ-lambda, and ι-iota), as they differ in the number and position of the ester sulfate groups on the repeating galactose units (Figure 2) [103]. Carrageenan is easily extractable in a few steps and has extensive industrial applications such as thickeners, stabilizers, gelling agents, and the development of biodegradable plastic. Carrageenan also has good barrier properties for preparing film packaging at a lower cost.

#### 6.2.2. Agar-Based Bioplastic

Agar and carrageenan play nearly the same functions. Agar thickens, stabilizes, and controls jellies’ viscosity in around 80% of production [104,105]. Agarose and agaropectin, two polysaccharides, are combined to form agar. Agarose gives things their ability to gel, while agaropectin gives them the ability to thicken (Figure 2).

### 6.3. Main Compounds of Bioplastics Produced from Green Macroalgae

Green macroalgae, or Chlorophyta, are prevalent in coastal waters and exhibit a greenish tint due to the significant content of chlorophylls a and b [17]. With cellulose, starch, and polysaccharides in abundance, green macroalgae emerge as a promising raw material for bioplastic production [17,106,107].

#### 6.3.1. Ulvan-Based Bioplastics

Ulvan, a sulfated heteropolysaccharide found in the cell walls of macroalgae in the order Ulvales, such as in *Ulva* and *Enteromorpha* spp., is composed of repeating disaccharide units including rhamnose, uronic acid, iduronic acid, and xylose. Notably, aldobiuronic acids, specifically ulvanobiouronic acid 3-sulfate type A3S and B3S, constitute the major repeating disaccharides. A3S comprises β-D-guluronic acid (1,4)-α-L-rhamnose-3-sulfate, while B3S consists of α-L-iduronic acid (1,4)-α-L-rhamnose-3-sulfate unit (Figure 2). Minor monosaccharides like xylose, glucose, and galactose are also present [108]. They can be used in packaging and in different biomaterial designs whose applications vary from drug delivery to wound dressing or bone tissue engineering [109,110].

#### 6.3.2. Starch-Based Bioplastic

Starch, a natural polymer found in green macroalgae, is a promising material for bioplastics due to its abundance, biodegradability, and compatibility with other biopolymers. It is a polysaccharide composed of two types of glucose units, namely, amylose and amylopectin [111]. Starch has several properties that make it suitable for the production of bioplastics, including its abundance, biodegradability, and compatibility with other biopolymers [112]. The structure and composition of algal starch can differ from plant starch. Algal starch tends to have a higher amylose content compared to plant starches like corn and potato starch [113]. The higher amylose can influence the material properties of the resulting bioplastic. Starch extracted from algae has been studied as a potential substitute for commercial corn starch in various applications, including the production of biodegradable materials as bioplastics [114]. Seaweed starch granules are small (1.7–7 µm) and have a high amylose content, indicating that they can yield high-quality bioplastics. Physicochemical characterization of *Ulva* starch granules has shown similarities to cereals starch, but with specific properties that make them suitable for applications such as emulsifiers, molecule carriers, functionalization, and bioplastics [115]. Additionally, the presence of algal starch in biofilms has been found to improve their barrier properties, making them more hydrophobic and enhancing their performance as biodegradable materials [112].

## 7. Processing of Macroalgae for Bioplastic Production

In the following, the production process of bioplastics from macroalgae will be discussed, including harvesting and processing of macroalgae, alginate, or carrageenan from the macroalgae, and formation of bioplastic.

### 7.1. Harvesting and Processing of Macroalgae

Macroalgae have been used for centuries in various applications, such as food, fertilizers, and cosmetics [14,116]. However, the use of macroalgae for bioplastic production is a relatively new concept [93]. Typically, macroalgae can be obtained from two main sources: the natural habitats of marine ecosystems and specialized aquaculture systems designed for cultivation. This diversity in acquisition methods underscores the versatility of macroalgae as a resource for various applications. Macroalgae are harvested using various methods, including handpicking and mechanical harvesting [117,118]. Handpicking is a labor-intensive process that involves manually collecting macroalgae from the seashore. This method is commonly used in small-scale operations and is unsuitable for large-scale production [118]. On the other hand, mechanical harvesting involves using boats equipped with specialized machinery to collect macroalgae from the water. This method is more efficient and can be used for large-scale production. However, mechanical harvesting can cause environmental damage and disrupt marine ecosystems [117,118]. After harvesting, macroalgae are washed to remove impurities and salt. The washed macroalgae are then dried to reduce moisture content and increase shelf life. Drying can be achieved using various methods, such as sun drying, air drying, or mechanical drying. Sun drying is the most common method used in small-scale operations, while mechanical drying is suitable for large-scale production [119].

### 7.2. Extraction of Polysaccharides from Macroalgae

The production of macroalgae bioplastic involves the extraction of polysaccharides from the macroalgae. Alginate and laminarin are extracted from brown macroalgae, while carrageenan and ulvan are extracted from red and green macroalgae, respectively [88,89,120].

#### 7.2.1. Extraction of Alginate

**Figure 3:**
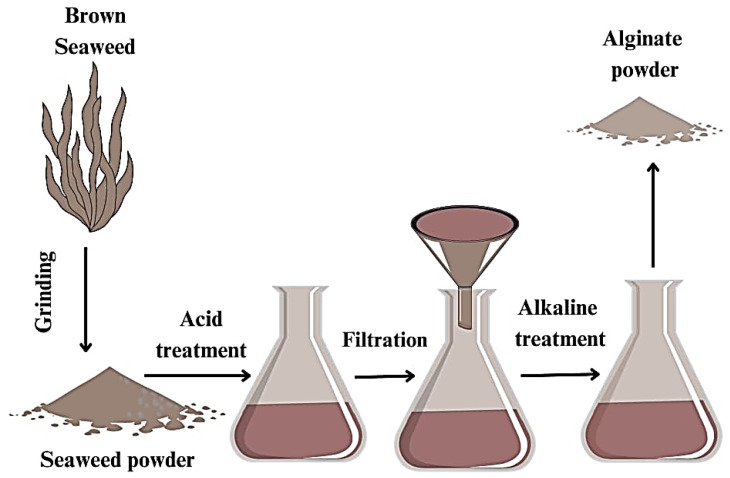
Extraction of alginate from brown macroalgae.

The extraction of alginate from macroalgae involves several steps. First, the dried macroalgae are ground into a powder. The powder is mixed with water and a mineral acid, such as hydrochloric acid, formaldehyde, or sodium hypochlorite solutions. The acid solution breaks down the cell walls of the macroalgae, releasing the alginate. After the acid treatment, the mixture is filtered to remove any impurities. The filtrate is then neutralized using an alkali solution, such as sodium hydroxide. The neutralization process forms a gel-like substance, which is then dried to form alginate powder [121,122,123], as expressed in Figure 3. Alginate films are typically produced by casting and solvent evaporation methods [124]. Ionic crosslinking of the alginate polymer with Ca^+2^ ions can significantly enhance the mechanical properties, barrier properties, and water resistance compared to films made with sodium alginate [125,126]. Common ionic crosslinking techniques include external gelation, internal gelation, interfacial gelation, and direct mixing of the crosslinking agent [127]. Covalent crosslinking using compounds like ferulic acid and citric acid has also been used to produce alginate films with improved thermal stability, flexibility, and transparency [128]. Another method is thermo-mechanical mixing and compression molding, which allows the blending of alginate with other thermoplastics and plasticizers like glycerol to improve the processability and flexibility of the bioplastic [129]. Key applications of alginate bioplastics include edible food coatings and packaging due to the polymer’s gas barrier properties, control over the release of bioactive compounds, and GRAS (Generally Recognized As Safe) status [87]. Alginate films have also been widely studied for biomedical uses such as wound dressings and drug delivery matrices [130,131]. Other applications include membranes for water treatment, agricultural mulch films, and flexible electronics [132,133,134].

#### 7.2.2. Extraction of Laminarin

**Figure 4:**
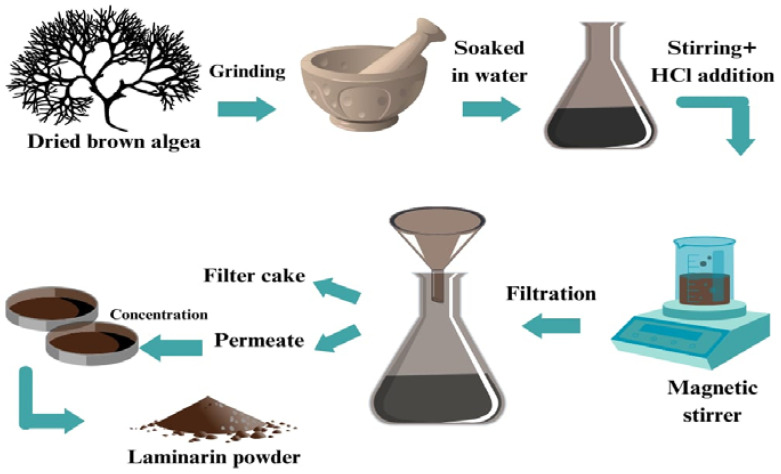
Extraction of laminarin from brown macroalgae.

Laminarin is a polysaccharide found in brown algae that can be extracted using various methods. The brown seaweed powder is soaked in water. The mixture is agitated or stirred to increase the extraction efficiency with the addition of HCl [135,136]. The mixture is then filtered using a mesh to obtain filter cake for alginate extraction and permeate for laminarin extraction. The laminarin solution is concentrated by evaporating the solvent using a rotary evaporator or a freeze-dryer. The concentrated laminarin solution is then purified, as shown in Figure 4. The extraction and purification of laminarin from brown algae might be a complex and time-consuming process, indicating a more experimental need to improve the process.

#### 7.2.3. Extraction of Ulvan

**Figure 5:**
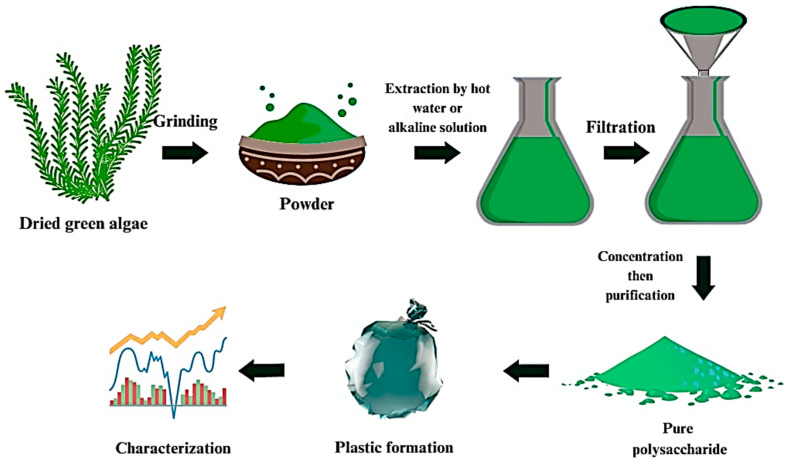
Extraction of ulvan from green macroalgae.

As shown in Figure 5, the general methodology for extracting ulvan from green macroalgae begins with collecting them from natural habitats and washing them thoroughly to remove any debris. Then, the washed macroalgae are ground to a fine powder using a grinder or blender. The resulting powder is sieved to obtain a uniform particle size. The following step is the extraction of ulvan by using hot water or a dilute alkali solution for several hours. The temperature and pH of the extraction medium are important factors that affect the yield and quality of the ulvan. After that, the extract is filtered to remove any insoluble particles, and the resulting solution is concentrated using techniques such as vacuum evaporation or freeze drying. Finally, the concentrated extract is purified to obtain pure ulvan and characterized using various techniques, such as Fourier transform infrared (FTIR) spectroscopy, nuclear magnetic resonance (NMR) spectroscopy, and gel electrophoresis, to determine its structure and properties [137,138].

#### 7.2.4. Extraction of Starch

**Figure 6:**
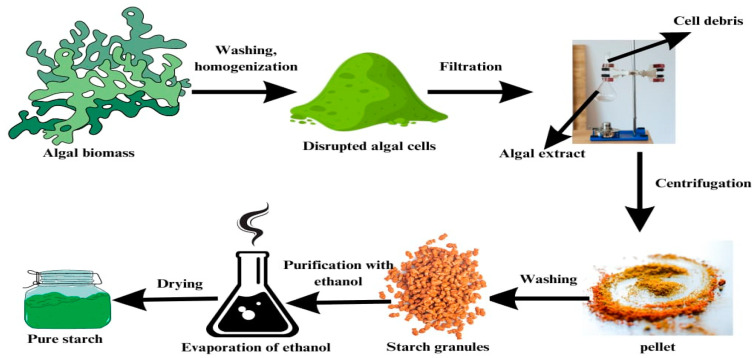
Extraction of starch from green macroalgae.

Extraction of starch from macroalgae begins with harvesting the green algae, washing the biomass several times with cold distilled water, and then homogenization. The mixture was filtered each time to remove large particles. The final filtrate was centrifuged to obtain a pellet that contained starch granules. The pellet was washed with ethanol for purification and to remove any related substances [115]. The ethanol was finally evaporated, and the pellet was dried and characterized (Figure 6).

#### 7.2.5. Extraction of Fucoidan

**Figure 7:**
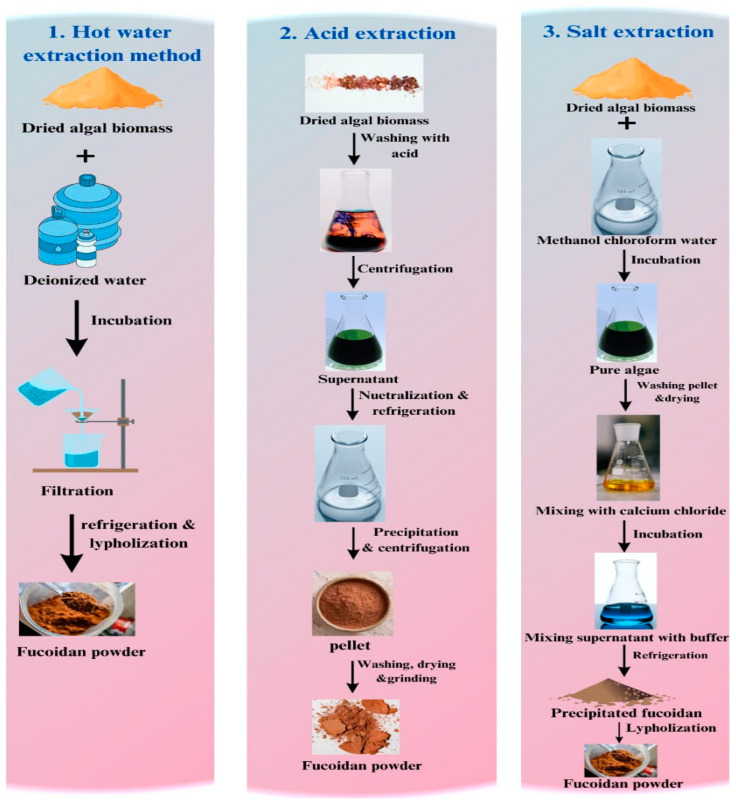
Extraction of fucoidan from brown macroalgae.

Fucoidan is a polysaccharide found in brown algae whose extraction methods are varied. The hot water extraction method involves dissolving algal biomass in deionized water, followed by incubation with agitation. The next steps are filtration, refrigeration, and lyophilization to form dry powder. The second method of extraction is acid extraction. In this method, dried algal biomass is washed with hydrochloric acid, then the supernatant is centrifuged and neutralized with an alkaline solution such as sodium hydroxide, followed by refrigeration. Ethanol was then added to precipitate fucoidan, which was then collected by centrifugation. The fucoidan pellet is washed using ethanol, dried, and ground to a fine powder. The third method is by using salt extraction. The dried algal biomass is washed with a mixture solution of methanol, chloroform, and water during stirring conditions to separate contaminations. The next step is washing the pellet with acetone and then drying it in a fume hood. The pellet is mixed with CaCl_2_ to extract fucoidan while incubated in stirring conditions. The supernatant is then mixed with a suitable buffer and refrigerated to precipitate fucoidan [139]. The mixture is centrifuged to collect fucoidan and washed using an ethanolic sodium iodine solution. Finally, lyophilization is used to obtain a water-soluble white powder of fucoidan (Figure 7).

#### 7.2.6. Extraction of Carrageenan

**Figure 8:**
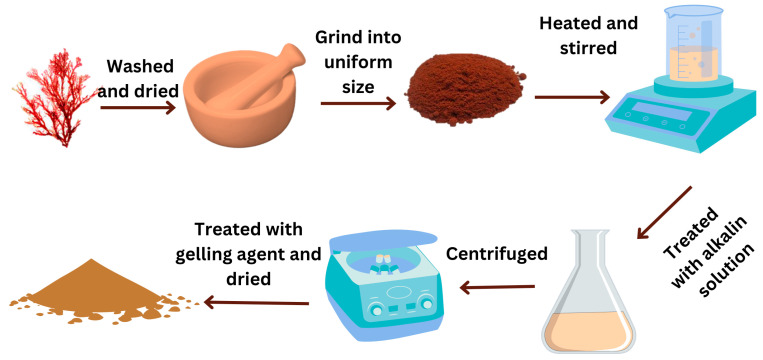
Carrageenan extract from red macroalgae.

The sun-dried red seaweeds were grounded into small particles or flakes and sieved to achieve uniformity. Carrageenan extraction can be easily performed using the hot water method (hydrolysis). The dried powders were soaked in- hot water (60–90 °C) for 2 h and treated with the alkaline solution at different concentrations to release carrageenan [140]. Carrageenan-rich extract was separated from the solid residue using vacuum filtration or centrifugation techniques. Isopropyl alcohol, or potassium chloride, was added as a gelling agent to precipitate carrageenan [140,141]. The precipitated carrageenan was thoroughly washed to remove impurities and dried under controlled conditions (Figure 8).

The methods employed in extracting and purifying the product result in refined or semi-refined carrageenan. The key distinction lies in the presence of cellulose, with semi-refined carrageenan retaining this component, while refined carrageenan boasts high purity by excluding cellulose. Consequently, additional extraction and purification steps become necessary to eliminate cellulose, contributing to an augmented overall production cost. However, selecting semi-refined products presents a cost-effective alternative to producing carrageenan-based bioplastics. It is worth noting that while semi-refined carrageenan can have robust gels, they may exhibit slightly less clarity compared to those derived from refined carrageenan [108].

#### 7.2.7. Extraction of Agar

**Figure 9:**
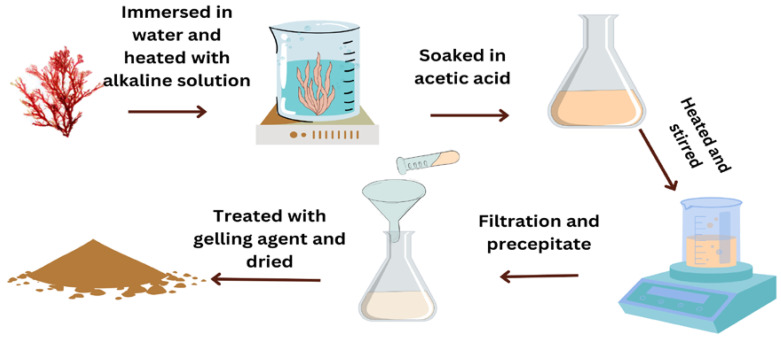
Extraction of agar from red algae.

The alkaline extraction method is a commonly used technique for the extraction of agar [142]. The grounded red seaweeds are immersed in water and heated with an alkaline solution like sodium hydroxide followed by acetic acid. This treatment helps in solubilizing agarose, the main component of agar. The mixture is heated and stirred continuously to facilitate extraction. The temperature and duration depend on the specific conditions and type of red algae used. After sufficient extraction, the mixture is filtered to separate the agar solution from the residue. A filter or cheesecloth removes solid particles, obtaining a clear liquid. The extracted agar solution is further treated with a gelling agent or chilled to induce agarose precipitation. Agarose can form a gel-like structure when cooled or with the addition of gelling agents. Common gelling agents include ethanol, isopropanol, or potassium chloride. The precipitated agarose gel is washed with water to remove impurities or traces of gelling agents. Finally, it is dried under controlled conditions to obtain agar flakes or powder (Figure 9).

### 7.3. Formation of Bioplastic

The formation of macroalgae bioplastic involves thermo-reversible gels, casting, compression molding, and extrusion-blowing techniques. Plasticizers like glycerol are often incorporated to improve flexibility [21]. The mixture is then heated and stirred to dissolve the polysaccharide powder. After the powder is dissolved, the solution is poured into a mold and allowed to cool and solidify. Ionic or covalent crosslinking of polymers such as alginate and carrageenan with calcium and other multivalent cations creates hydrogel films with tailorable physical properties. Blending different algal polysaccharides allows for tuning bioplastic characteristics for diverse applications ranging from food packaging to medical devices [143,144]. Table 1 lists some examples of bioplastic processing methods.

## 8. Mechanical and Physical Characteristics of Bioplastics

Generally, bioplastics can be classified according to their physical, optical, mechanical, morphological, thermal, antioxidant, antibacterial, and biodegradable properties and characterization data. The films’ thickness, solubility, moisture content, water vapor permeability, and water vapor transmission rate are examples of their physical attributes. Light transmittance, opacity, and transparency are examples of optical characteristics. Young’s modulus, elongation at break, and tensile strength are examples of mechanical properties. The study of morphology can be examined by X-ray diffraction analysis (XRD), atomic force microscopy (AFM), field emission scanning electron microscopy (FESEM or SEM), and FTIR spectroscopy. Thermal gravimetric analysis (TGA) and differential scanning calorimetry (DSC) are two examples of thermal characteristics. The temperature differential at which a material can withstand heat is known as its thermal resistance [131]. The ability of a material to permit a vapor, e.g., water vapor or any other gas, to pass through it is known as vapor permeability. The faster water and vapor can move through a substance, the greater its permeability value [108]. Biodegradability is another feature that is frequently sought for food packaging [139]. Each bioplastic has a distinct life cycle or length of complete biodegradation due to the large diversity of bioplastics obtained from different sources. Therefore, these changes are based on the biodegradation conditions and waste management systems in each site. To define the worth of an ideal bioplastic product, mechanical characteristics must be evaluated, such as tensile strength (TS), elongation at break (EAB), thermal resistance (TR), and water vapor permeability. The characteristics and attributes of the seaweed bioplastic films were indorsed by many studies compared to petroleum-based films [6,104,139]. Seaweeds derived from alginate, agar, carrageenan, and cellulose proved exceptional film-forming properties with easy processing protocols. Besides their practical mechanical properties and shorter shelf life, seaweed bioplastic would be a better casual to combat elevated plastic pollution. Seaweed bioplastics are naturally eco-benign and degradable in soil. However, seaweed bioplastics still have weakness points, including being fragile and rigid. To overcome this weakness, natural seaweed bioplastic plasticizer materials are added [157].

### 8.1. Tensile Strength (TS) and Elongation at Break (EAB) Properties of Bioplastics

TN analysis is applied to determine the force required to break down a specimen and how far it will stand stretching. The TS data are particularly important to a variety of industries to assess the production of bio-based plastic goods [158]. TS is usually measured in Mega Pascal (MPa) units using the CMT-10 Computer Control Electronic Universal Testing Machine [158]. The minimum standard value of TS of bioplastics was stated to be above 0.39 MPa by the Japanese industrial standards in 1975 (Japanese Standards Association 2 1707, 1975). Elongation at break (EAB) denotes the ability of a film to elongate from its starting length to the breaking point [159]. A matrix composed of natural polymers expresses the capacity to withstand changes of shape without breaking formation. The EAB standard was set at 10–50% based on the percent elongation of the plastic (Japanese Standards Association 2 1707, 1975). According to the data from [158], the increase in EAB value using different plasticizers was followed by a decrease in the TS of the bioplastic. Biopolymer films with TS in the range of 10–100 MPA and EAB > 10% are considered to have good mechanical properties [160]. Seaweed bioplastics can be made from hydrocolloids, such as alginate, carrageenan, and ulvan [135]. Plasticizer additives will change the mechanical and physical properties of the produced plastic to be more elastic and less rigid. Lim et al. [104] investigated the physical properties of alginate biofilm developed from the brown alga *Sargassum siliquosum.* During film processing, alginate was mixed with sago starch, sorbitol, and CaCl_2_ solution. The results indicated that the biofilm developed from a mixture of alginate powder (2 g), sorbitol (15% *w*/*w*), and 75% *w*/*w* of CaCl_2_ presented substantial physical properties, including TS, EAB, water vapor permeability, and water solubility, which were comparable to HDPE or PP plastics. Paula et al. [131] examined the physical properties of glycerol-plasticized edible films made from κ-carrageenan, ι-carrageenan, and alginate hydrocolloids. κ-carrageenan films exhibited higher TS and elasticity, higher water permeability, and lower opacity than ι-carrageenan, while alginate films showed higher transparency [131]. Carrageenan and alginate hydrocolloids form a rigid and stable gel matrix in the presence of cations, particularly Ca^2+^. Films obtained from sodium alginate with 1–3% (*w*/*v*) CaCl_2_ solution displayed increased TS and elongation properties while decreasing opacity [110]. Compared to carrageenan and alginate films, fabricated agar films have a lower TS and water vapor permeability. Yet, agar films showed improved elasticity and twice the EAB value of κ-carrageenan films [133]. Due to their valuated viscosity and gelling properties, seaweed hydrocolloids are widely used as gelling, stabilizers, and/or thickening agents in the food, pharmaceuticals, and cosmetics industries.

### 8.2. Thickness Property of Bioplastics

The thickness of films protects from gas permeability and resists pests; therefore, it is important for the protection of food products. The standard thickness of commercial polyethylene packaging films is between 0.025 and 0.075 mm [160]. Bioplastic thickness can be measured using a digital micrometer instrument or a manual screw micrometer [158]. Carrageenan bioplastic films, with the addition of 20% PEG (polyethylene glycol), sorbitol, or glycerol as a plasticizer, showed an average thickness increase of 13.1, 15.53, and 18.05%, respectively [158]. Plasticizers of hygroscopic properties can absorb moisture; this causes the plastic polymer to contain more water and increase in thickness. However, the results showed that different types of plasticizers had no significant effect (at *p* > 0.05) on the resulting thickness changes. Theoretically, increasing the concentration, number, and/or types of materials used for making bioplastic films will further increase their solid content and viscosity, resulting in increased thickness [128]. The area of the mold and the type of additive used also affect the thickness of the bioplastic product [129].

### 8.3. Thermal Resistance (TR) Property of Bioplastics

One important aspect of bioplastics’ performance is their TR, which refers to how well they maintain their shape and structure properties when exposed to heat. The thermal properties of macroalgae-derived bioplastics are influenced by many factors such as the type of polysaccharide, molecular weight, and degree of polymerization. TR of bioplastics is an important factor in determining their suitability for various applications. Higher molecular weight and degree of polymerization generally resulted in increased TR. When exposed to high temperatures, bioplastics undergo thermal degradation through the breakdown of the polysaccharide chains, leading to a loss of mechanical properties and structural integrity. For example, on k-carrageenan extract from *Kappaphycus alvarezii*, Sudhakar et al. [139] evidenced good physical, mechanical, and thermal strength of the developed bioplastic films. In another investigation performed by Hanry and Surugau [143] bioplastics developed from *K. alvarezii* whole biomass crosslinked with commercial sodium alginate had a degradation peak at 270 °C when the percentage of sodium alginate was zero. By increasing the sodium alginate percentage to 100% relative to *K. alvarezii* biomass, the degradation peak decreased to 223 °C. As shown in the same example, degradation of glycosidic bonds in cellulosic parts of *K. alvarezii* biomass, decarboxylation, decarbonylation, and hydration of commercial alginate additives occurred above 170 °C to 400 °C [143]. Among green seaweeds, ulvan polysaccharide extracts showed a unique film-forming property. These extracts exhibit high TR and mechanical strength, which coexitwith the characteristics of optimal bioplastics [135]. Researchers are exploring various methods to improve the thermal stability of bioplastics from macroalgae. These include blending with other polymers, incorporating plasticizers, and modifying processing techniques.

### 8.4. Plasticizers Addition for Improving Bioplastic Properties

Plasticizer addition can significantly influence the mechanical properties of bioplastics. Plasticizers are organic materials, which are blended with biopolymers to enhance their flexibility, elasticity, and processability [158]. The efficiency of a plasticizer is related to its ability to make the target material soft and flexible for expanded limitations of shelf life and product use [139]. The plasticization reduces the stiffness of the 3D structure of bioplastic film, decreases the relative number of polymer–polymer interactions, and allows its deformation without breaking [140]. An increase in plasticizer concentration may enhance the flexibility of the films, but may potentially decrease TS. Common plasticizers are glycol and sorbitol. Glycerol absorbs more water than sorbitol, thus affecting the hydrophilic tendency of bioplastics [160]. The selection of plasticizers is critical, as it affects the bioplastic product characteristics, compatibility, toxicity, and cost. Table 2 lists the main physical properties of some bioplastics synthesized from seaweed biomass or their extracted polymers, with/out plasticizer addition. The application of plasticizers can increase the density and thickness of the bioplastic. The data from the literature analyzed by [158] showed that the thickness of plasticized films increased with increasing plasticizer content, irrespective of their type. Rasheed and co-authors [161] verified that sorbitol-based bioplastic films are significantly thicker than glycerol-based bioplastic ones. Sorbitol increased the molecular weight of the films over glycerol thereby supporting the thickening of the developed films. Moreover, alginate-based bioplastic films were recommended as acceptable and profitable raw materials for industries [161]. The optimized alginate films showed a TS of 33.90 MPa, EAB of 3.58%, water vapor permeability of 2.63 × 10^−10^ g Pa^−1^ s^−1^m^−1^, and water solubility of 33.73%. Manufactured bioplastic films with thicknesses ranging from 0.050 to 0.054 mm were suitable for commercial food packaging [162].

The TS property of bioplastic also changes after plasticizer addition. Most studies reported a decrease in the TS of bioplastics due to the ability of plasticizers to reduce the intermolecular bonds of the polymer and facilitate the migration of water vapor, so that the bonds and the TS of the plastic polymer are becoming weaker while the EAB property becomes stronger [158]. The whole biomass of *Kappaphycus* sp. seaweed was mixed with commercial sodium alginate at different ratios. When more alginate was added, the TR and TS declined while elasticity and water barrier properties increased. The addition of commercial alginate disrupted the homogeneity of the *Kappaphycus* sp. blends resulting in a drop in TS and hydrophobicity of the biofilms [162]. The physicochemical properties of κ-carrageenan films extracted from *Eucheuma cottonii* seaweed were studied by Balqis et al. [160]. The κ-carrageenan films were combined with different concentrations and types of plasticizers including, glycerol, sorbitol, and polyethylene glycol-300 (PEG-300) in the range of 10–60%. Glycerol-plasticized films produced the thickest films as glycerol absorbs more moisture than other plasticizers. As the plasticizer concentration increased, the TS value of the films declined due to the enlarged spatial distance between the polymer molecules caused by the added plasticizers [141]. There was no significant variation in TS between different types of plasticizers. The EAB of k-carrageenan films increased significantly with increasing the plasticizer content. Plasticizers increase the mobility of polymer chains, resulting in more elastic and flexible films. Sorbitol-plasticized films had the lowest EAB compared to other developed films. Sorbitol films had the minimal ability to absorb water, so the polymer’s mobility was reduced and its physical properties met the standards of packaging films [160]. K-carrageenan extracted from *K. alvarezii* seaweed was shown to be suitable for the manufacturing of bioplastic films. In this work, the bioplastic film produced from *K. alvarezii* shows superior physical and mechanical capabilities when combined with PEG-300 (3% *w*/*v*) as a plasticizer [157] (Table 2). Kappa-carrageenan films blended with 4% PEG had a higher TS than those of 3% and 5% PEG. The TS values of the films were standard for commercial synthetic bioplastic. According to the EAB test, concentrations of 4% and 5% of films had lower values than 3% of films [157]. This could be related to the use of entire seaweed biomass, which decreased the other compounds in EAB. This also indicated that a precise combination of polymer and plasticizer concentration is required to achieve the optimum EAB strength of a certain bioplastic film. Kanagesan and co-authors [159] created high-quality green bioplastic films from alginate (Table 2). Various film formulations of alginate blend with inverted sugar (IS) were developed. The control film without IS was stiff and slightly brittle, but bioplastic with IS blends was flexible. The presence of IS at lower concentrations showed higher TS values. However, raising the IS concentration resulted in a considerable drop in the TS and sticky films [159]. In a study by El-Sheekh et al. [163], a bioplastic film made from *Halimeda opuntia* with glycerol as a plasticizer and polyvinyl alcohol (PVA) as a film-forming polymer showed excellent physical and mechanical capabilities. The seaweed-based bioplastic was flexible, smooth, and rapidly biodegradable. The blend ratio of seaweed/PVA affected the biofilm properties with the best criteria for the ratio of 3:1, *w*/*v* bioplastic film. The developed bioplastic sheets were appropriate for food and non-food packaging industries, as well as medication capsules, and can help to reduce environmental pollution.

## 9. Biodegradability of Macroalgae Bioplastics

Biodegradation naturally occurs due to the mineralization of materials by microorganisms (such as bacteria, algae, and fungi) action. The methods of biodegradation include fouling, erosion, hydrolysis, and pigment coloration via diffusing into the polymers and degrading of leaching components to finally produce CO_2_ and H_2_O [20]. The biodegradation of bioplastic polymers is related to their chemical nature and the persistent surrounding environmental factors. In water environments, the complete degradation of the water-soluble monomers and oligomers by microorganisms could be confirmed by a BOD biodegradation test in a laboratory setting. Alternatively, the buried in the soil test was mostly performed to assess plastic degradation in soil environments [63]. Conventional petroleum-based plastics take significantly more time to degrade than bioplastics made from organic or biotic sources. The degradation of bioplastics differs in different environments (soil, aquatic habitat, and compost system). However, bioplastics might be biodegradable in some environments better than others. Bioplastic-composted soil increases the soil microbiological content and fertility and raises the crop yield [64]. Alternatively, the aquatic environment is the highest in terms of being prone to plastic pollution. The bioplastic degradation in freshwater and marine habitats exhibits a slower rate than that in soil environments, composting, or anaerobic digestion [64]. This may be due to the lack of microbial diversity in the aqueous ecosystems.

Biodegradable plastics are not directly biobased plastics. The biodegradability of bioplastic polymers depends on the chemical structure of the polymers and their interaction with environmental factors, such as moisture, temperature, acidity, etc. [164]. Composting allows microorganisms to convert organic matter into CO_2_, H_2_O, inorganic compounds, and biomass. This is considered an excellent EOL option for biodegradable bioplastics without evolving visible or toxic remains. The composting process is mostly suitable for degrading food-contaminated plastic packaging to develop composts that can be used for soil amendment as fertilizers [64]. In addition, compost is the most appropriate approach for bioplastic degradation, followed by soil and aquatic decomposing environments. Compost (or anaerobic digestion) can affect some biodegradable plastics, which soil cannot. Bioplastics derived from seaweed biomass or polysaccharide polymers verified the criteria for active biodegradability and low environmental persistence. Despite having a similar monomer structure, starch and cellulose differ in their polymeric chain architecture. Cellulose-based biopolymers have common usage due to their strength, high endurance, and biodegradability. Among tested biopolymers, the highest and quite fast biodegradability percentage was recorded for cellulose-based bioplastics by 80–100% after 100 days of burying in compost environments [165]. A 47-day composting experiment found that 97 ± 7% of cellulose mineralized after standard composting methods were applied. Starch-based bioplastic degradation in non-industrial composite conditions after 9 weeks of composting has been observed [64]. Bioplastic degradation in composting settings is influenced mainly by temperature and the chemical composition of bioplastics. In composting studies, temperatures above 55 °C resulted in amorphous polymers that are more hydrophilic [166]. In general, starch- and cellulose-based bioplastics are hydrolyzable and so are booked as an EOL option by biodegradation. However, caution should be demonstrated during biodegradation of such polymers to confirm complete digestion. Also, to prevent hazardous side effects such as micro- and nanoplastics development or the outflow of pollutants during degradation processing [64]. A feasible method for green bioplastics production from alginates of brown seaweeds in Sabah, Malaysia, was recommended by Kanagesan et al. [139]. The addition of 5% inverted sugar as a plasticizer to 6% alginate produced a bioplastic material with good standard properties including TS and biodegradability. The bioplastic started to degrade on day 1 (by 25 %) and completed degradation (by 100 %) on day 4 compared to the synthetic control plastic, which was undegradable. Similarly, about half of the alginate-based bioplastic (46.51%) extracted from *Padina pavonica* was completely biodegraded after 45 days of being buried in the soil [167]. In another study by El-Sheekh et al. [163], a thin bioplastic film was produced by optimizing the ratio of polyvinyl alcohol (PVA) to seaweed *Halimeda opuntia* biomass. The bioplastic film showed better physical and mechanical properties at 3:1 of *H. opuntia*/PVA ratio with a biodegradability percentage of 47.03% after 30 days in clay. It was also degradable in sand by 36.68% and in compost by 100% after 30 and 5 days, respectively. Although they are the most subjected to plastic contamination, bioplastic degradation in seawater or freshwater is slower than biodegradation in soil, composting, and anaerobic digestion environments. This was related to the specific parameters (such as temperature, pH, nutrients, microbial density, and biodiversity) of these habitats, as well as the bioplastics’ composition. It was reported that a starch-based bioplastic degraded by 1.5% (at 25 °C, 90 days) under marine and freshwater environments [165]. The starch-based shoppers showed a weight loss of 69% within 236 days depending on the bioplastic features and the environmental conditions. It should be noted that aquatic environments might degrade some biodegradable plastics, but never to be used as EOL site fill. A possible approach to alleviate the bioplastic pollution in aquatic environments (where most plastic waste occurs) is using algal biomass [166]. As microorganisms, microalgae are a potential candidate for benign plastic degradation in aquatic habitats. They do not contain endotoxins like bacteria or need organic carbon sources under photoautotrophic conditions [164]. They can grow on an artificial substrate, colonize it, and start the biodegradation process using exopolysaccharides and degrading ligninolytic enzymes [166].

Although the biodegradability of biopolymers would result in reducing the plastic threat and emerging contaminants, some difficulties need to be overcome. Pollution from non-compostable plastics and bioplastics, long degradation time, and how additives could affect biodegradation rates are all issues of concern for proper recycling of bioplastics and their LCA [164]. Recently, micro and nanoscale plastics as well as plastic leachates have entered different aquatic environments through wastewater discharges. However, few data were available about wastewater discharges releasing or shedding bioplastics. In this context, it is necessary to introduce biocatalysts—enzymes, microorganisms, and candidate genes—for the degradation of plastics by growing microbes. These microbes can selectively depolymerize bioplastic waste into its constituent monomers or other value-added products [63].

## 10. Examples of Products Made from Bioplastics

**Figure 10:**
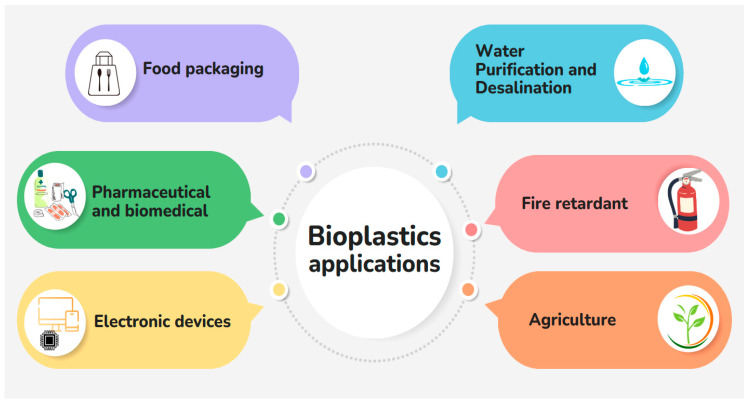
Different applications of bioplastic from seaweeds.

Bioplastics are renewable, decomposable, and have a little carbon footmark, and thus are a promising substitute for traditional plastic [20,164]. Figure 10 and Table 3 describe some examples of products made from bioplastic.

## 11. Challenges and Limitations of Macroalgae Bioplastic Production

**Figure 11:**
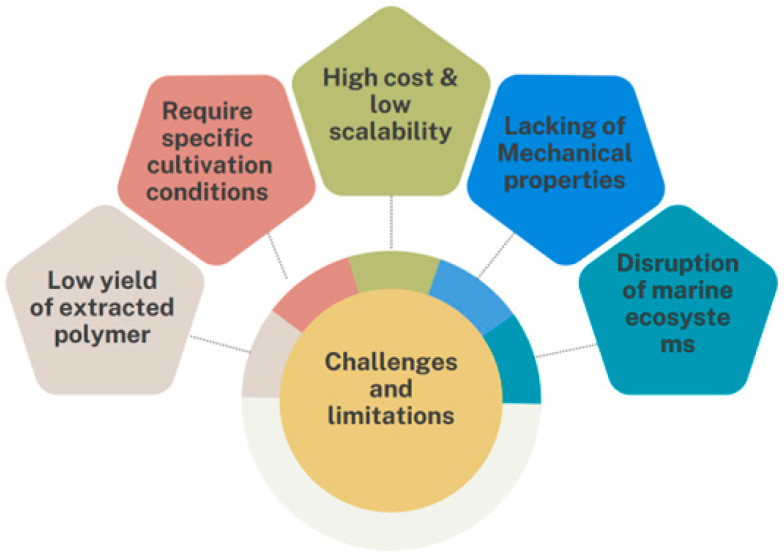
Challenges and limitations of bioplastic production.

Macroalgal-based bioplastics have the potential to displace conventional plastics derived from fossil fuels, lessening the harm that plastic waste causes to the environment. However, to reach its maximum potential, macroalgae bioplastic manufacturing must overcome a number of obstacles and restrictions (Figure 11). The low yield of polymer extraction is a significant obstacle in the manufacturing of macroalgae bioplastics. Since most macroalgae have little polymer, it is challenging to extract enough for the manufacturing of bioplastics. Practically, it is challenging to optimize extraction techniques because the polymer content of macroalgae changes based on the species, growing circumstances, and solvents used [143]. Another difficulty in producing macroalgae bioplastics is macroalgae farming. The production of macroalgae for sustainable biomass needs particular environmental parameters, like temperature, light, and nutrient availability. These variables, which change based on the region and season, also affect the macroalgal growth rate. Additionally, the enormous land or water expanses needed for macroalgae production may limit its scalability [185]. The macroalgae bioplastic production faces other limitations in terms of mechanical properties and durability. While bioplastics produced from macroalgae are biodegradable, they often lack the optimum mechanical properties required for durable products for long-term use [191]. The potential applications of the macroalgae-based polymer developed are limited due to its hydrophilic nature, poor heat resistance, and tendency to break easily. Consequently, techniques for adjusting the polymer’s characteristics must be developed in order to improve its durability and mechanical qualities [185]. Furthermore, there are financial and scalability challenges associated with the production of macroalgae bioplastic. The cultivation, extraction, and polymer modification of macroalgae come at a comparatively higher cost than that of fossil plastics. The lack of experience, the ability to extract and change the polymer on a wide scale, and the availability of suitable growing regions limit the scalability of macroalgae bioplastic manufacturing [15].

Another difficulty associated with macroalgae bioplastic production is the potential impact on marine ecosystems [143]. Macroalgae play an important role in marine ecosystems, providing habitats and food sources for marine organisms. The large-scale cultivation of macroalgae for bioplastic production may have negative impacts on marine ecosystems, including the loss of biodiversity and disruption of marine food chains. Therefore, it is necessary to develop sustainable cultivation methods that minimize the negative impact on marine ecosystems [164]. An additional crucial challenge is the recycling of biodegradable plastics, which is still risky and low in research. Emissions of hazardous gases during landfilling of bioplastics and the uncertainty of complete biodegradability in open environmental systems are also issues for further consideration.

## 12. Conclusions

Bioplastic production from macroalgae-derived polysaccharides has the potential to offer a sustainable alternative to traditional petroleum-based plastics. Macroalgae are a highly abundant and renewable resource that can be harvested without harming the environment. The use of macroalgae for bioplastic production also offers several benefits, including reduced carbon emissions and decreased dependence on non-renewable resources. However, the development of macroalgae-based bioplastics is still in the early stages, and several challenges need to be addressed. These include the development of efficient and cost-effective extraction methods, as well as the optimization of bioplastic production processes. Still in its infancy, the production of bioplastics derived from macroalgae has a number of obstacles that must be overcome, which emphasizes the present techniques for producing algal polymer’ blend bioplastics of upgraded properties using genetic engineering and new biotechnology techniques. In addition, there is a need for further research on the properties, applications, and biodegradability of macroalgae-based bioplastics. The products released from the degradation of bioplastics such as CO_2_, micro-or nanoplastics leaches, and other harmful byproducts must be considered for their effect on the environment. The efficiency in polymer biodegradation by microbial enzymes (including microalgal enzymes) and colonization on bioplastic substrata as a primary step in plastic biodegradations are still obstacles. Despite these challenges, the potential benefits of macroalgae-based bioplastics are significant, and continued research and development in this field could lead to a more sustainable and environmentally friendly future.

## Figures and Tables

**Table 1:** Some examples of bioplastic processing methods.

Bioplastic	Processing Methods	Details	References
Alginate	Casting	Sodium alginate is dissolved in water, glycerol or other plasticizers can be added, the solution is cast onto a surface like glass or plastic, and the solvent evaporates, leaving an alginate film.	[124]
	External gelation	Films are first cast from sodium alginate, as above. Then, the dried films are immersed in a CaCl_2_ solution, which diffuses into the film, crosslinks the alginate chains, and improves film properties. Time in calcium solution can be optimized to control the extent of crosslinking.	[126,128]
	Compression molding	Alginate and plasticizers are premixed and heated to increase viscous flow, then pressed between mold plates at specific pressures and temperatures. Allows incorporation of thermoplastics.	[129]
Fucoidan	Blending	Fucoidan lacks gel-forming ability alone, so it is blended with polymers like alginate or chitosan. They can be dissolved together in aqueous solutions before casting films.	[145]
Laminarin	Thermo-reversible gelation	Laminarin gelatinized when cooled below 40 °C, which can produce films.	[146]
	Blending	Laminarin blending with other polymers like glycerol, chitosan, or crosslinking improves moisture and mechanical properties.	[147]
Carrageenan	Casting	Carrageenan powder is dissolved in water along with plasticizers, then poured or spread onto a surface for solvent evaporation.	[148]
	Ionic crosslinking	Divalent cations like Ca^+2^ are added to ι-carrageenan solutions, or K+ is added to κ-carrageenan, which induces helix formation and gelation. Crosslinking cations can be optimized.	[149]
	Blending	Blending with glycerol increases the plasticizer content, and the tensile strength decreases, but the elongation break increases, water vapors transmission, and oxygen transmission.	[150]
	Casting	Hot agar solution is poured onto a warm surface above its gel point. As water evaporates, hydrogen bonding between agar chains allows film formation. Silicone casting surfaces can help prevent sticking.	[151]
Agar	Thermo-reversible gelation	Agar powder is added to water and heated to 90–100 °C to dissolve. Upon cooling to 32–40 °C, the agar chains transition from random coils to double helices, inducing gelation.	[152]
	Blending	Blending agar with silky, clear, and firm glycerol for both commercial and algal agar. They lose weight by heating but still have the same texture. Algal agar has a higher water-holding capacity than commercial agar. They can be maintained for nine months.	[150]
Ulvan	Casting	Similar to carrageenan, ulvan powder is dissolved in water, plasticizers are added, and then cast into films.	[153]
	Thermo-reversible gelation	Aqueous ulvan solutions form gels upon heating and cooling cycles between 25 and 80 °C. Gel strength depends on ulvan molecular weight.	[154]
Starch	Casting	Algal starch is dissolved in water with plasticizers like glycerol and cast into films similarly to other polysaccharides.	[155]
	Heating in water	Starch can be gelatinized by heating in excess water, and then retrograded by cooling to make films.	[156]
	Blending	Ulvan and starch show good miscibility and interact synergistically when blended. Glycerol plasticization further improves green algal starch film properties.	[115]
	Compression molding	Heat and pressure can be used to produce thermoplastic starch bioplastic objects.	[144]

**Table 2:** Main properties of bioplastic: thickness, tensile strength, and elongation at break.

Bioplastic	Plasticizer	Thickness (mm)	Tensile Strength (TS) (MPa)	Elongation at Break (EAB) (%)	Ref.
Alginate	ــــــــــــ	0.023	ــــــــــــ	ــــــــــــ	[161]
	Sorbitol	0.23: 0.676			
	Glycerol	0.113: 0.27			
Whole biomass *Kappaphycus* sp. + sodium alginate	Glycerol	0.054	7.91 ± 0.45	ــــــــــــ	[162]
Kappa-carrageenan	ـــــــــــــ	0.03153	69.69	3	[160]
	Glycerol	0.05627	39	13	
	Sorbitol	0.05567	41	11	
	PEG-300	0.05533	39	17	
Kappa-carrageenan (3%)	PEG-300	0.806	9.13	7.1	[157]
Kappa-carrageenan (4%)		0.097	13.78	4.72	
Kappa-carrageenan (5%)		0.079	12.90	2.72	
Alginate (6%)	ــــــــــــ	ــــــــــــ	14.96	0.86	[159]
	Inverted sugar		2.13	17.44	
*Halimeda opuntia: PVA* films in ratios	Glycerol				[163]
0.5:1		0.037	147.5	420.3	
1:1		0.015	127.8	363.7	
1.5:1		0.020	157.3	440	
2:1		0.025	173.4	408.5	
3:1		0.028	181.2	436.6	

**Table 3:** Different applications of seaweed-based bioplastics.

Field of Application	Polysaccharide	Composite or Transformed Form	Specific Application	Ref.
Food packaging and coatings	Crude Alginate	Calcium alginate	Calcium alginate films are rich in co-extracted phlorotannins polyphenols	[87]
	Carrageenan and Alginate	Carrageenan and calcium alginate	Films with promoted properties	[168]
	Alginate and Carrageenan	Alginate with two different types of carrageenan (κ- and ι-carrageenan)	Films with different physical properties	[149]
	Carrageenan and Ulvan	Semi-refined carrageenan and ulvan	Edible composite films with antioxidant properties	[169]
	Alginate	Graphene oxide and zinc oxide as an electrically conducting and active filler in alginate films and sepiolite for compatibility	Edible films with an electrical conduction property	[170]
	Alginate	Alginate/gellan	Water-resistant films as a coat on paper cups for hot drinks by spraying method	[171]
Pharmaceutical and biomedical	Alginate	Human elastin-like polypeptide with alginate and cross-linked with curcumin and CaCl_2_	Wound dressing	[172]
	Ulvan and Chitosan	Ulvan/chitosan layer-by-layer films	Membranes used for neural implants and devices	[168]
	Ulvan	Ulvan was cross-linked using 1,4-Butanediol diglycidyl ether	Wound dressing for controlled release of a steroid antiinflammatory drug	[130]
	Alginate and Carrageenan	Sodium alginate and κ-carrageenan with potassium ions	Wound dressings with good swelling and bioactivity	[168]
	Carrageenan and Agar	κ-carrageenan was blended with agar	Biocompatible wound dressing	[173]
	Chitosan and Fucoidan	Chitosan/fucoidan porous film	Wound dressing	[174]
	Fucoidan	Collagenfucoidan blend films	Tissue regeneration	[175]
	Chitosan and Ulvan	Chitosan/ulvan composite membranes are further crosslinked by tripolyphosphate and glycerol	Wound dressing	[176]
Water purification and desalination	Alginate	Calcium alginate	Films for the removal of heavy metals from an aqueous solution	[134]
	Cellulose and Carrageenan	Anionic nanocomposite using cellulose, carrageenan, and TiO_2_	Nanocomposite catalyzed the removal of methylene blue by photodegradation	[177]
	Alginate	Alginate and activated carbon	Membranes removed the analgesic drug diclofenac from solutions	[178]
	Carrageenan	κ-carrageenan into polyvinylidene fluoride membrane	Water separation from methyl orange dye solution	[168]
	Alginate	Alginate and bacterial cellulose	Separate ethanol/water mixtures	[168]
	Chitosan and Carrageenan	Multiwalled carbon nanotubes/chitosan/ι-carrageenan membrane	Remove heavy metals	[179]
	Alginate	Graphene oxide in sodium alginate membrane	Water desalination by pervaporation	[180]
Agriculture	Alginate	Alginate crosslinking by Ca^2+^	Films are carriers of plant nutrients	[133]
	Alginate	Na-alginate	Na-alginate mulching coatings for stimulation of plant growth	[181]
Electronic devices	Alginate	Graphene/calcium alginate thin films	Electromagnetic interference shielding	[132]
	Alginate	Ti_3_C_2_Tx/Ca alginate films	Aerogel film provides electromagnetic interference shielding	[182]
	Alginate	Alginate hydrogel-polyacrylamide composite embedded with silver flakes	Material for electricity conduction	[183]
	Chitosan and Carrageenan	Chitosan/ι-carrageenan composite films with H_3_PO_4_ and poly(ethylene glycol)	Electrical double-layer capacitors as separators and electrolytes	[184]
	Alginate	Lithium alginate with poly(ethylene oxide) and polyacrylamide	Batteries	[185]
	Alginate	Sodium alginate/attapulgite nanofiber	Membrane as a potential separator in lithium-ion batteries	[186]
	Alginate and Cellulose	Calcium alginate and cellulose	Membranes with good performance as a separator in batteries	[187]
	Alginate and Carrageenan	Alginate and κ-carrageenan	Methanol fuel cells	[188]
Fire retardant	Carrageenan	Carrageenan fibres	Fibers with good flame-retardant properties	[189]
	Agar and Alginate	Agar and sodium alginate with boric acid	Flame-retardant composite films	[168]
	Chitosan and Alginate	Soluble chitosan, sodium alginate, and Cu^+2^	Coat for polyester fabric	[168]
Other application	Carrageenan	Carboxymethyl κ-carrageenan, CMC, and ammonium iodide	Polymer electrolyte films	[190]

## Data Availability

All data generated or analyzed during this study are included in this published article.
